# Processing and Technology of Dairy Products: A Special Issue

**DOI:** 10.3390/foods9030272

**Published:** 2020-03-03

**Authors:** Hilton Deeth, Phil Kelly

**Affiliations:** 1 School of Agriculture and Food Sciences, The University of Queensland, Brisbane 4072, Australia; 2 Teagasc Food Research Centre Moorepark, Fermoy, P61 C996 Co. Cork, Ireland; philk51@hotmail.com

When this Special Issue was launched, we cast the net widely in terms of the subject matter we considered suitable for the papers. We stated that papers on “well-established unit operations such as heat treatments and membrane separation in addition to emerging technologies” would be welcomed. The seven papers accepted do, indeed, cover a range of topics including UHT milk, proteolytic digestion, membrane technologies, cheese and yogurt. Three papers [1,2,3] involve aspects of the beneficial uses of proteolytic enzymes, two [4,5] involve the use of membrane technology in cheese making, while two deal with the role of ingredients—raw milk in the UHT paper [6] and apricot fiber in the yogurt paper [7]—in product quality. All in all, the papers demonstrate the breadth of ongoing research for an industry based on just one raw material, milk.

Each submission explores innovative approaches by the respective authors in their quest to push the boundaries of scientific and technological understanding. Some examples are illustrated below: Chamberland et al. [4] address the question of whether one should chose a 0.1 μm pore size MF or 10 kDa molecular weight cut-off ultrafiltration (UF) membranes for cheese milk standardization. The authors found that the UF, rather than the MF membrane, scored better in terms of lower running (energy and membrane) costs. In a related study, the sensory quality of hard, high-cooked cheese processed from milk, preconcentrated 1.9 fold by reverse osmosis, is shown by Taivosalo et al. [5] to be largely unaffected. Heat stability represents an important field of study in dairy science, and in this issue, readers have the opportunity to consider the approach of Karlsson et al. [6], who undertook a full factorial designed study on the role of key milk components on the stability of UHT milk. Crude preparations of apricot fiber (Karaca et al. [7]) were demonstrated as a novel ingredient with the capacity to confer functional benefits during yogurt processing.

As editors of this special issue, we find it appropriate to reflect on how well the scientific originality of the reviewed manuscripts scored against sustainability criteria. The two membrane-based papers concerned with either protein standardization [4] or milk pre-concentration [5] impact cheesemaking efficiency directly through yield improvements, shorter manufacturing processes and increased manufacturing capacity (without the need for extra cheese vat capacity). Chamberland et al. [4] go one step further by differentiating between closely matched permeating UF and MF polymeric membranes in favor of UF, because of its lower energy usage and membrane replacement costs. Protein hydrolysis is not typically associated with an opportunity to fractionate whey protein, except Sáez et al. [3] identified an opportunity during a particular set of incubation conditions in which the breakdown of α-lactalbumin (α-la) could be delayed. Before putting in place a strategy to recover undigested α-la, Sáez et al. [3] identified a number of shortcomings during application of a range of non-thermal and thermal methods of inactivating the enzyme-containing hydrolysate. Chief among these was the unexpected amount of heat-induced aggregation taking place among peptides and undigested protein in the whey protein hydrolysates which ruled out subsequent fractionation efforts. Suddenly, what was perceived to be a very elegant, sustainable, dual enzymatic hydrolysis/fractionation process was ground to a halt and is pending the next stage of development. The positive interaction when a plant-based ingredient is shown to be functional in yogurt [7} is evidence of how synergies may be harnessed when a holistic approach is adopted via food ingredient combinations, i.e., plant and dairy can co-exist in formulated foods where they can be enjoyed for both the pleasure of eating as well as the health benefits that they bring to the consumer.

It is important to recognize the valuable dissemination contribution that ***foods*** is making through this Special Issue: Processing and Technology of Dairy Products, given its achievement of a high impact factor, its commitment to a rapid turnaround of peer-reviewed manuscripts before publication, and its accessibility to a wide audience.

## References

[1] Estrada O., Ariño A., Juan T. (2019). Salt Distribution in Raw Sheep Milk Cheese during Ripening and the Effect on Proteolysis and Lipolysis. Foods.

[2] Gutiérrez-Méndez N., Balderrama-Carmona A., García-Sandoval S.E., Ramírez-Vigil P., Leal-Ramos M.Y., García-Triana A. (2019). Proteolysis and Rheological Properties of Cream Cheese Made with a Plant-Derived Coagulant from Solanum elaeagnifolium. Foods.

[3] Sáez L., Murphy E., FitzGerald R.J., Kelly P. (2019). Exploring the Use of a Modified High-Temperature, Short-Time Continuous Heat Exchanger with Extended Holding Time (HTST-EHT) for Thermal Inactivation of Trypsin Following Selective Enzymatic Hydrolysis of the β-Lactoglobulin Fraction in Whey Protein Isolate. Foods.

[4] Chamberland J., Mercier-Bouchard D., Dussault-Chouinard I., Benoit S., Doyen A., Britten M., Pouliot Y. (2019). On the Use of Ultrafiltration or Microfiltration Polymeric Spiral-Wound Membranes for Cheesemilk Standardization: Impact on Process Efficiency. Foods.

[5] Taivosalo A., Kriščiunaite T., Stulova I., Part N., Rosend J., Sõrmus A., Vilu R. (2019). Ripening of Hard Cheese Produced from Milk Concentrated by Reverse Osmosis. Foods.

[6] Karlsson M.A., Lundh Å., Innings F., Höjer A., Wikström M., Langton M. (2019). The Effect of Calcium, Citrate, and Urea on the Stability of Ultra-High Temperature Treated Milk: A Full Factorial Designed Study. Foods.

[7] Karaca O.B., Güzeler N., Tangüler H., Yaşar K., Akın M.B. (2019). Effects of Apricot Fibre on the Physicochemical Characteristics, the Sensory Properties and Bacterial Viability of Nonfat Probiotic Yoghurts. Foods.

